# Multi-Dimensional Spectrum-Effect Relationship of the Impact of Chinese Herbal Formula Lichong Shengsui Yin on Ovarian Cancer

**DOI:** 10.3390/molecules22060979

**Published:** 2017-06-13

**Authors:** Yanhong Wang, Yang Li, Yan Zhang, Guan Feng, Zhixin Yang, Qingxia Guan, Rui Wang, Fengjuan Han

**Affiliations:** 1 College of Pharmacy, Heilongjiang University of Traditional Medicine, Harbin 150040, China; wangyanhong@hljucm.net (Y.W.); zhangyan19870327@foxmail.com (Y.Z.); guanfeng@hljucm.net (G.F.); yangzhixin@hljucm.net (Z.Y.); gqxwyb@163.com (Q.G.); wrdx@sina.com (R.W.); 2 Disha Pharmaceutical Group, Qingdao 266100, China; leeyoung_alex@163.com; 3 The First Affiliated Hospital, Heilongjiang University of Chinese Medicine, Harbin 150040, China

**Keywords:** Lichong Shengsui Yin, multi-dimensional fingerprints, anti-ovarian cancer, spectrum-effect relationships

## Abstract

Lichong Shengsui Yin (LCSSY) is an effective and classic compound prescription of Traditional Chinese Medicines (TCMs) used for the treatment of ovarian cancer. To investigate its pharmacodynamic basis for treating ovarian cancer, the multi-dimensional spectrum-effect relationship was determined. Four compositions (I to IV) were obtained by extracting LCSSY successively with supercritical CO_2_ fluid extraction, 75% ethanol reflux extraction, and the water extraction-ethanol precipitation method. Nine samples for pharmacological evaluation and fingerprint analysis were prepared by changing the content of the four compositions. The specific proportions of the four compositions were designed according to a four-factor, three-level L_9_(3^4^) orthogonal test. The pharmacological evaluation included in vitro tumor inhibition experiments and the survival extension rate in tumor-bearing nude mice. The fingerprint analyzed by chromatographic condition I (high-performance liquid chromatography-photodiode array detec tor，HPLC-PDA) identified 19 common peaks. High-performance liquid chromatography-photodiode array detector-Evaporative Light-scattering Detector (HPLC-PDA-ELSD )hyphenated techniques were used to compensate for the use of a single detector, and the fingerprint analyzed by chromatographic condition II identified 28 common peaks in PDA and 23 common peaks in ELSD. Furthermore, multiple statistical analyses were utilized to calculate the relationships between the peaks and the pharmacological results. The union of the regression and the correlation analysis results were the peaks of X_5_, X_9_, X_11_, X_12_, X_16_, X_18_, Y_5_, Y_8_, Y_12_, Y_14_, Y_20_, Z_4_, Z_5_, Z_6_, and Z_8_. The intersection of the regression and the correlation analysis results were the peaks of X_11_, X_12_, X_16_, X_18_, Y_5_, Y_12_, and Z_5_. The correlated peaks were assigned by comparing the fingerprints with the negative control samples and reference standard samples, and identifying the structure using high-performance liquid chromatography-mass spectrometry detector(HPLC-MS). The results suggested that the pharmacodynamic basis of LCSSY on anti-ovarian cancer activities were germacrone, furandiene, β-elemene, calycosin-7-glucoside, ononin, epimedin B, icariin, ginsenoside Rc, astragaloside, ginsenoside Rd, astragaloside II, and some unknown components.

## 1. Introduction

Traditional Chinese medicines (TCMs) have played an increasingly significant role in protecting human health and fighting against disease in China for thousands of years [1]. TCMs have a long history of clinical use and are effective in the treatment of numerous diseases, especially various incurable diseases [2]. Following the increased incidence of cancer and chronic diseases, most therapies with chemical drugs still do not yield satisfactory outcomes in patients, and TCMs are widely accepted by billions of people all around the world. Compared with chemical drugs, the advantages of TCMs are that they have multi-target, multi-level, and multi-composition characteristics. This means that the beneficial efficacy of TCMs is determined by the synergy between multiple compositions. However, an overabundance of ingredients can cause difficulties in investigating the mechanisms involved, identifying effective substances, and controlling the quality of TCMs. To promote the development of TCMs, numerous investigations and various methods have been devised to solve these problems [3,4,5]. Of these methods, the spectrum-effect relationship method has become a research hotspot in TCM studies.

The spectrum-effect relationship is a scientific method based on the fingerprint of TCM, which determines the correlations between fingerprint and activity. The method can clarify the pharmacodynamic basis and establish evaluation methods to control TCM quality [6]. Establishment of the fingerprint, the pharmacodynamic evaluation and the data processing are important factors in spectrum-effect relationship studies. The fingerprints of TCMs can be obtained using a variety of equipment and instruments, such as HPLC [7,8], high performance thin layer chromatography (HPTLC) [9], gas chromatography-mass spectrometry (GC-MS) [10], infrared spectroscopy (IR) [11], high performance capillary electrophoresis (HPCE) [12], and other new technology [13,14,15]. However, none of these describe the entire chemical information of TCMs [16]. Therefore, multidimensional and multivariate fingerprinting was proposed to achieve better fingerprints. This is not a new invention and does not use new equipment, but utilizes two or more sets of equipment associated with two or more detectors to obtain as much information as possible from the fingerprints of TCMs. This method is more appropriate for compound prescriptions of TCMs.

Lichong Shengsui Yin (LCSSY) composed of Curcumae Rhizome, Sparganii Rhizome, Astragali Radix, Ginseng Radix et Rhizoma, Fritillariae Thunbergii Bulbus, Cervi Cornu Pantotrichum, Epimedii Folium, and Hirudo, as an effective and classic compound prescription of TCMs, is derived from Li Chong Wan in *Yi Xue Zhong Zhong Can Xi Lu* which is well known for treating many gynecological diseases. Many pharmacodynamics experiments have been carried out to verify the therapeutic efficacy of LCSSY in ovarian cancer [17,18,19,20,21,22,23], but the therapeutic mechanism and pharmacodynamic basis are unclear. This study aims to investigate the pharmacodynamic basis of LCSSY for treating ovarian cancer using the spectrum-effect relationship method.

## 2. Results and Discussion

### 2.1. Results of the Orthogonal Compatibility Anticancer Experiment

**Figure 1:**
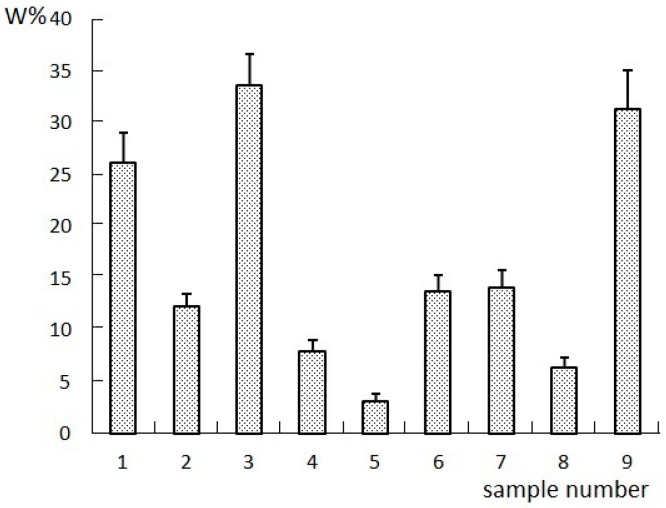
Results of W% (the cell proliferation inhibition rate) in vitro tumor inhibition experiments in each group.

**Figure 2:**
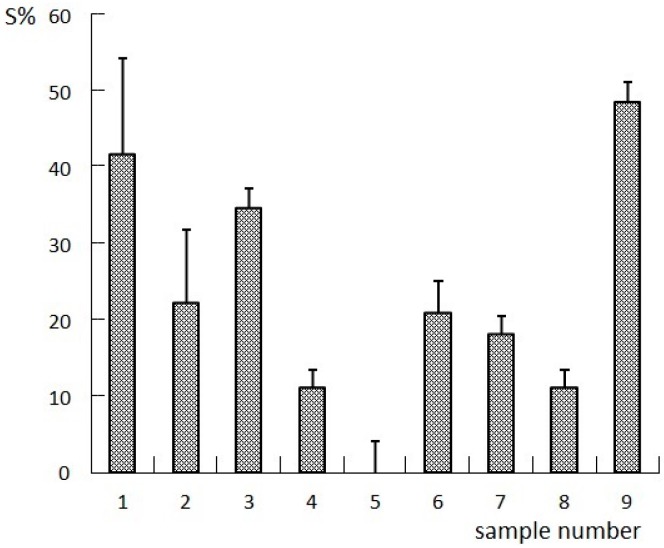
Results of S% (the survival extension rate) in tumor-bearing nude mice.

The Chinese herbal formulas for spectrum-effect relationship studies are generally divided into different batches, different extraction methods, or different combinations [6]. Different combinations refer to the herbs, extracts, or compositions in an orthogonal compatibility combination. In this study, four compositions (I to IV) were obtained by extracting LCSSY successively with supercritical CO_2_ fluid extraction, 75% ethanol reflux extraction, and the water extraction-ethanol precipitation method. Nine samples for pharmacological evaluation and fingerprint analysis were prepared by changing the content of the four compositions. The specific proportions of the four compositions were designed according to a four-factor, three-level L_9_(3^4^) orthogonal test. The pharmacological evaluation included in vitro tumor inhibition experiments and survival extension rate in tumor-bearing nude mice. The results of the anticancer experiment are shown in Figure 1 and Figure 2.

### 2.2. Results of the HPLC Experiment

Due to the complex chemical composition of Chinese herbal formulas, and the large differences in physical and chemical properties, obtaining the “spectrum” which reflects the entire chemical information is the key in the spectrum-effect relationship. LCSSY is comprised of *Curcumae Rhizome*, *Sparganii Rhizome*, *Astragali Radix*, *Ginseng Radix et Rhizoma*, *Fritillariae Thunbergii Bulbus*, *Cervi Cornu Pantotrichum*, *Epimedii Folium*, and *Hirudo.* There are also volatile oils, flavonoids, and saponins in LCSSY. In order to obtain more comprehensive chemical information on LCSSY, two methods of fingerprint analysis were established.

#### 2.2.1. Fingerprints Analyzed by Chromatographic Condition I (HPLC-PDA)

The results of the methodology validation showed that the relative standard deviation(R.S.D.) of the relative retention time and relative peak area of the characteristic for precision were less than 0.32% and 1.5%, for stability were less than 0.42% and 3%, and for repeatability were less than 0.64% and 2.5%, respectively. These results demonstrate that the method used for the HPLC fingerprint analysis was stable and reliable.

#### 2.2.2. Fingerprints Analyzed by Chromatographic Condition II (HPLC-PDA-ELSD)

HPLC-PDA-ELSD hyphenated techniques were used to compensate for the use of a single detector. The results of the methodology validation showed that the R.S.D. of the relative retention time and relative peak area of the characteristic for precision were less than 0.5% and 1.5% in PDA and less than 1% and 1.8% in ELSD, for stability were less than 0.2% and 2% in both PDA and ELSD, and for repeatability were less than 1% and 2.5% in PDA and less than 1.3% and 3% in ELSD, respectively. These results demonstrate that the method used for the HPLC fingerprint analysis was stable and reliable.

#### 2.2.3. Determination of Fingerprints

**Figure 3:**
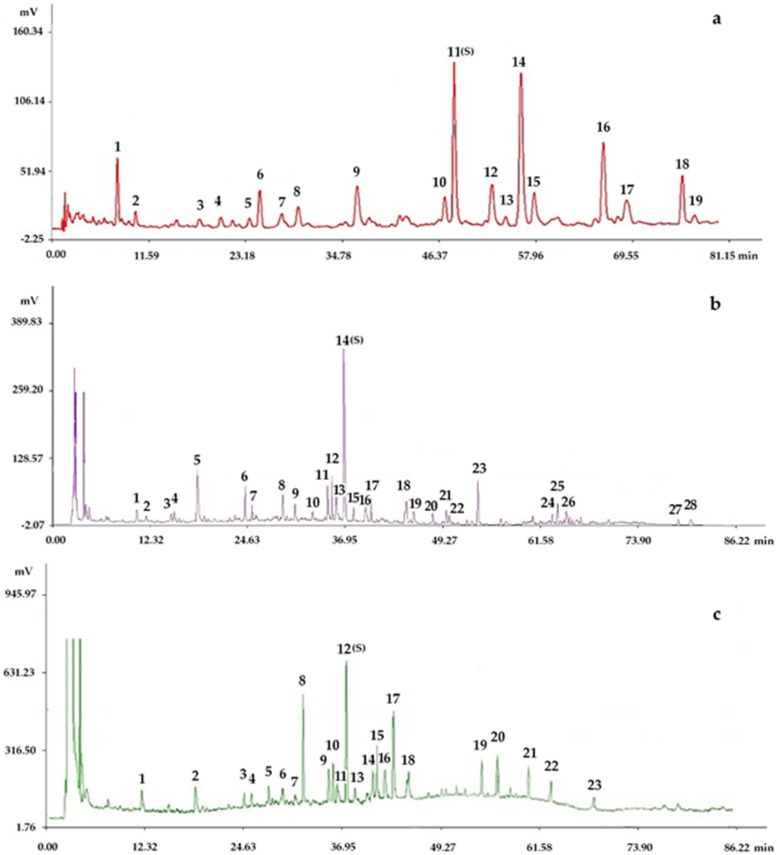
Representative fingerprints of the nine samples designed by the L_9_(3^4^) orthogonal test: (**a**) the fingerprint analyzed by chromatographic condition I (HPLC-PDA), in which 19 peaks with large areas and good segregation were obtained as “common peaks”; (**b**) the fingerprint analyzed by chromatographic condition II (HPLC-PDA), in which 28 peaks with large areas and good segregation were regarded as “common peaks”; (**c**) the fingerprint analyzed by chromatographic condition II (HPLC-ELSD), in which 23 peaks with large areas and good segregation were regarded as “common peaks”.

The representative fingerprints of the nine samples designed by the L_9_(3^4^) orthogonal test are shown in Figure 3.

### 2.3. The Analysis of Spectrum-Effect Relationships

To avoid repeated analysis of some of the same compositions in PDA and ELSD [24] analyzed by chromatographic condition II, fourteen peaks were regarded as redundant peaks and excluded from the statistical calculations. Thus, 28 characteristic peaks marked as Y_1_ to Y_28_ in the PDA fingerprint analyzed by chromatographic condition II (HPLC-PDA), 9 characteristic peaks marked as Z_1_ to Z_9_ in the ELSD fingerprint analyzed by chromatographic condition II (HPLC-ELSD), and 19 characteristic peaks marked as X_1_ to X_19_ in the fingerprint analyzed by chromatographic condition I (HPLC-PDA) were selected to investigate the spectrum-effect relationships between the components and the pharmacological effect. Finally, the peak areas of nine prescriptions associated with the cell proliferation inhibition rate of W and survival extension rate of S formed a 9*56 data matrix for subsequent statistical analysis. The data processing methods used in the “spectrum-effect relationship” studies mainly included correlation analysis, regression analysis, and principal component analysis. Different methods have different focus points. Therefore, one or more data processing methods are commonly used in combination.

#### 2.3.1. Regression Analysis

##### ENTER Method

The ENTER regression equation of W and S was respectively established by analyzing the independent variable of peak area and the dependent variables of W and S. Eight peaks were included in the equations which were X_5_, X_11_, X_12_, X_16_, X_18_, Y_5_, Y_12_, and Z_6_, the equations of which were:

W_1_ = 0.023X_5_ + 0.006X_11_ − 0.003X_12_ − 1.027 × 10^−6^X_16_ + 0.017X_18_ − 0.008Y_5_ + 0.031Y_12_ − 0.002Z_6_ − 50.90; S_1_ = 0.003X_5_ − 0.020X_11_ + 0.007X_12_ − 0.008X_16_ − 0.023X_18_ − 0.004Y_5_ + 0.003Y_12_ + 0.011Z_6_ − 38.833. (1)

Scientific notation The residuals statistics of Durbin–Watson reflected the independence between residuals in the range of 2 ± 1.5. The determination coefficient was 0.912 in W_1_ and 0.881 in S_1_, and both the residuals statistics were 2.382. The results of variance analysis demonstrated statistical significance (*p* < 0.05).

##### STEPWISE Method

The STEPWISE regression equations of W and S were respectively established by analyzing the independent variable of peak area and the dependent variables of W and S. Four peaks were retained in the equations: X_11_, X_12_, Y_5_, and Z_4_, the equations of which were:

W_2_ = −0.005X_11_ + 0.007X_12_ − 0.008Y_5_ + 0.003Z_4_ − 145.66; S_2_ = 0.007 Z_5_ + 706.429. (2)

The determination coefficient was 0.896 in W_2_ and 0.901 in S_2_, and both residuals statistics were 3.012. The results of variance analysis demonstrated statistical significance (*p* < 0.05).

#### 2.3.2. Correlation Analysis

**Figure 4:**
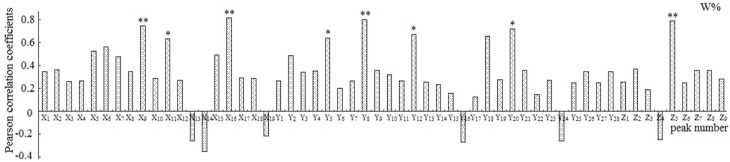
Pearson correlation coefficients between the variables and pharmacologic data W%. **: *p* < 0.01, *: *p* < 0.05; W% was the cell proliferation inhibition rate.

**Figure 5:**
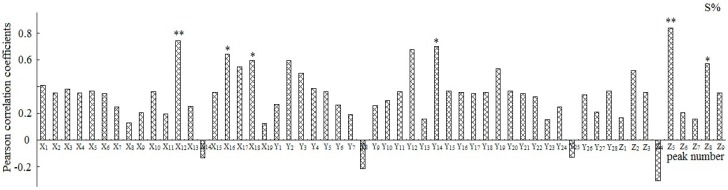
Pearson correlation coefficients between the variables and pharmacologic data S%. **: *p* < 0.01, *: *p* < 0.05; S% was the survival extension rate.

Each of the peaks was respectively analyzed with the W and S, and the relationships between the peak area and the pharmacological results were reflected by Pearson’s correlation coefficient (Figure 4 and Figure 5). The results demonstrated that the peaks X_9_, X_11_, X_16_, Y_5_, Y_8_, Y_12_, Y_20_, and Z_5_ to W and the peaks X_12_, X_16_, X_18_, Y_14_, Z_5_, and Z_8_ to S showed statistical significance (*p* < 0.05).

#### 2.3.3. Integration of the Analytical Results

The union of the ENTER and STEPWISE results in the regression were the peaks of X_5_, X_11_, X_12_, X_16_, X_18_, Y_5_, Y_12_, Z_4_, Z_5_, and Z_6_. The results in the correlation analysis were the peaks X_9_, X_11_, X_12_, X_16_, X_1__8_, Y_5_, Y_8_, Y_12_, Y_14_, Y_20_, Z_5_, and Z_8._ The union of the regression and the correlation analysis results were the peaks of X_5_, X_9_, X_11_, X_12_, X_16_, X_18_, Y_5_, Y_8_, Y_12_, Y_14_, Y_20_, Z_4_, Z_5_, Z_6_, and Z_8_. The intersection of the regression and the correlation analysis results were the peaks of X_11_, X_12_, X_16_, X_18_, Y_5_, Y_12_, and Z_5_.

#### 2.3.4. Assignments of the Correlated Peaks

**Figure 6:**
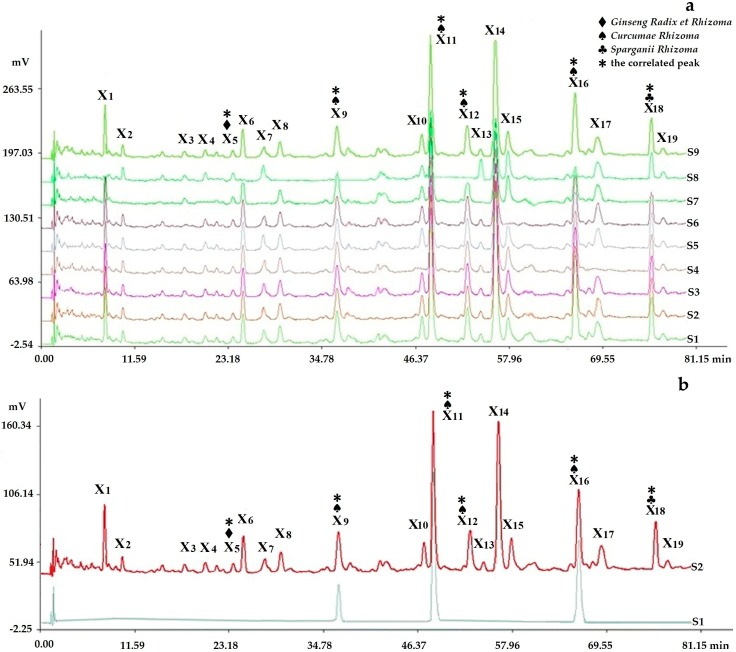
Chromatogram comparing the fingerprints analyzed by chromatographic condition I (HPLC-PDA) with negative control samples and reference standard samples: (**a**) S1–S8 were respectively the negative sample without *Hirudo*, without *Epimedii Folium*, without *Cervi Cornu Pantotrichum*, without *Fritillariae Thunbergii Bulbus*, without *Ginseng Radix et Rhizoma*, without *Astragali Radix*, without *Sparganii Rhizoma*, and without *Curcumae Rhizoma*, in which peak *5* was from *Ginseng Radix et Rhizoma*; peaks 9, 11, 12, and 16 were from *Curcumae Rhizoma*; peak 18 was from *Sparganii Rhizoma* by comparing with the negative control solutions (S1–S8); (**b**) S2 and S1 were respectively the fingerprints of the sample solution and reference standard solution, in which three peaks were respectively identified by comparing with the reference standard substance: peak 9, germacrone; peak 11(S), furandiene (reference peak); peak 16, β-elemene.

**Figure 7:**
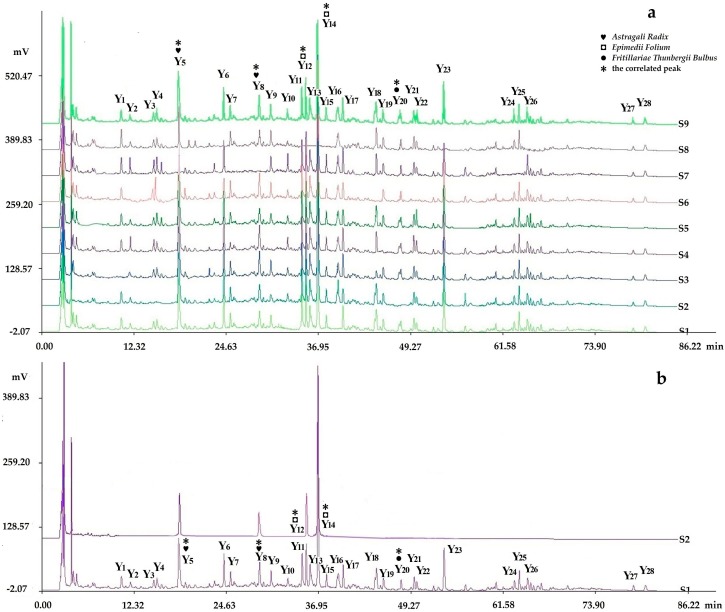
Chromatogram comparing the fingerprints analyzed by chromatographic condition II (HPLC-PDA) with negative control samples and reference standard samples: (**a**) S1–S8 were respectively the negative sample without *Cervi Cornu Pantotrichum*, without *Fritillariae Thunbergii Bulbus*, without *Hirudo*, without *Ginseng Radix et Rhizoma*, without *Curcumae Rhizoma*, without *Sparganii Rhizoma*, without *Astragali Radix*, and without *Epimedii Folium*, in which peaks 5 and 8 were from *Astragali Radix*; peaks 12 and 14 were from *Epimedii Folium*; and peak 20 was from *Fritillariae Thunbergii Bulbus* by comparing with the negative control solutions (S1–S8); (**b**) S2 and S1 were respectively the fingerprints of the reference standard solution and sample solution, in which peaks 8, 12, and 14(S) were identified as ononin, epimedin B, and icariin (reference peak) by comparing with the reference standard substance.

**Figure 8:**
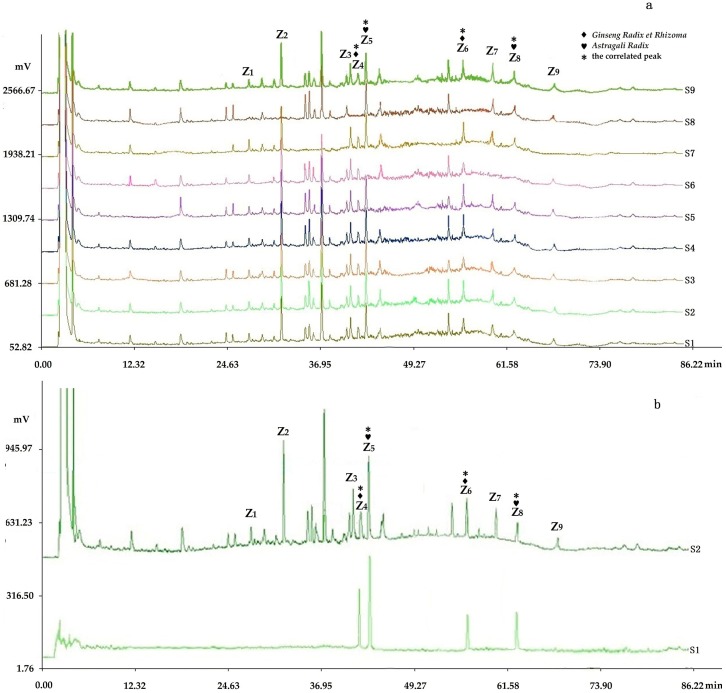
Chromatogram comparing the fingerprints analyzed by chromatographic condition II (HPLC-ELSD) with negative control samples and reference standard samples: (**a**) S1–S8 were respectively the negative sample without *Hirudo*, without *Curcumae Rhizoma*, without *Cervi Cornu Pantotrichum*, without *Fritillariae Thunbergii Bulbus*, without *Sparganii Rhizoma*, without *Astragali Radix*, without *Epimedii Folium*, and without *Ginseng Radix et Rhizoma*, in which peaks 4 and 6 were from *Astragali Radix*; peaks 5 and 8 were from *Ginseng Radix et Rhizoma* by comparing with the negative control solutions (S1–S8); (**b**) S2 and S1 were respectively the fingerprints of the sample solution and reference standard solution, in which four peaks were respectively verified by comparing with the reference standard substance: peak 4, ginsenoside Rc; peak 5, astragaloside; peak 6, ginsenoside Rd; peak 8, astragaloside II.

The results of the chromatograms comparing the fingerprints with negative control samples and reference standard samples (Figure 6, Figure 7 and Figure 8) and the structural identification of the correlated peaks analyzed by HPLC-MS (Table 1) showed that X_5_ was an unknown component from *Ginseng Radix et Rhizoma*; X_9_ was germacrone from *Curcumae Rhizoma*; X_11_ was furandiene from *Curcumae Rhizoma*; X_12_ was an unknown component from *Curcumae Rhizoma*; X_16_ was β-elemene from *Curcumae Rhizoma*; X_18_ was an unknown component from *Sparganii Rhizoma*; Y_5_ was calycosin-7-glucoside from *Astragali Radix*; Y_8_ was ononin from *Astragali Radix*; Y_12_ was epimedin B from *Epimedii Folium*; Y_14_ was icariin from *Epimedii Folium*; Y_20_ was an unknown component from *Fritillariae Thunbergii Bulbus*; Z_4_ was ginsenoside Rc from *Ginseng Radix et Rhizoma*; Z_5_ was astragaloside from *Astragali Radix*; Z_6_ was ginsenoside Rd from *Ginseng Radix et Rhizoma*; and Z_8_ was astragaloside II from *Astragali Radix*.

The above results suggest that the pharmacodynamic basis of the anti-ovarian cancer activities of LCSSY were germacrone, furandiene, β-elemene, calycosin-7-glucoside, ononin, epimedin B, icariin, ginsenoside Rc, astragaloside, ginsenoside Rd, astragaloside II, and some unknown components.

Germacrone and furandiene are the main indices for the quality control of zedoary turmeric oil. It was reported that germacrone can inhibit the proliferation of human ovarian cancer A2780 cells, colon cancer HCT, and nasopharyngeal carcinoma KB3 [25], and furandiene can inhibit the proliferation of many types of tumor cells, such as HL60 leukemia cells [26], and human hepatocellular carcinoma cells [27].

Numerous studies have reported that β-elemene has broad-spectrum anticancer effects, such as in ovarian cancer [28], glioblastoma cells [29], lung cancer [30,31], liver cancer, breast cancer, and brain cancer. Elemene can not only induce cell cycle arrest of different types according to different types of tumor cells, but also fights against drug-resistance.

Astragaloside is recognized as the main effective compound of Astragali Radix. It was reported that astragaloside could inhibit tumor growth in lung cancer [32,33]. It was reported that astragaloside II showed strong potency in increasing 5-fluorouracil cytotoxicity toward 5-fluorouracil-resistant human hepatic cancer cells Bel-7402/FU and may be a potential adjunctive agent for hepatic cancer chemotherapy [34]. Ginsenoside Rd can significantly inhibit the proliferation of human cervical cancer HeLa cells [35] and both gastric and breast cancer cells [36]. It was found that ginsenoside Rc can induce c-fos in MCF-7 human breast carcinoma cells at both the mRNA and protein levels [37].

Icariin has broad anticancer effects, such as in breast cancer [38], hepatoma [39], gastric cancer [40], melanoma [41], and ovarian cancer [42]. To date, there are few data on the anticancer effects of calycosin-7-glucoside, ononin, and epimedin B. The present study indicated the potential activity of these components, which requires further research.

The results mentioned in the above reports were in accordance with the results in the present study.

## 3. Materials and Methods

### 3.1. Reagents and Materials

All raw herbs comprising LCSSY, such as Curcumae Rhizome, Sparganii Rhizome, Astragali Radix, Ginseng Radix et Rhizoma, Fritillariae Thunbergii Bulbus, Cervi Cornu Pantotrichum, Epimedii Folium, and Hirudo were supplied by the First Affiliated Hospital, Heilongjiang University of Chinese Medicine (Harbin, China). Professor Huifeng Sun (College of Pharmacy, Heilongjiang University of Traditional Medicine, Harbin, China) authenticated these herbs and voucher specimens were deposited in the College of Pharmacy, Heilongjiang University of Traditional Medicine (Harbin, China).

SKOV3 tumor cell lines were purchased from Jiangsu Qishi Biological Science and Technology Co. Ltd. (Shanghai, China), incubated in fresh complete culture solution composed of 90% RPMI 1640, 10% fetal bovine serum, and 1% myllicin, and were then adjusted to yield approximately 10^7^ CFU/mL in each fresh complete culture solution.

Wistar rats (mean weight 200 ± 20 g, mean age 6 weeks) were bought from the Safety Evaluation Center of Heilongjiang University of Chinese Medicine (Harbin, China), and nude mice (mean weight 16 ± 2 g, mean age 4–6 weeks) inoculated with SKOV3 tumor cells were bought from Beijing Weitong Lihua Co. Ltd. (Beijing, China). Both the rats and nude mice were bred and the experiments were carried out in the Safety Evaluation Center of Heilongjiang University of Chinese Medicine (Harbin, China) according to the current national legislation on animal experiments. All animals were female and were randomized to the different treatment groups. The experimental protocol was approved by the Animal Ethics Committee of Heilongjiang University of Chinese Medicine and conformed to the National Institute of Health and Nutrition Guide for Care and Use of Laboratory Animals.

HPLC-grade acetonitrile and methanol were from Sigma-Aldrich (St. Louis, MO, USA). Methanol, ethanol, formic acid, and all other reagents were AR grade from Shanghai Wujin Chemical Regent Factory (Shanghai, China). Water was obtained from Watsons (Hong Kong, China).

### 3.2. Sample Preparation

A prescription volume of LCSSY was extracted by supercritical CO_2_ to obtain Composition I under the conditions of 30 Kpa extraction pressure and 50 °C extraction temperature, and was resolved at 6 Kpa and 50 °C with a SFE Device (Nantonghua’an Company, Nantong, China). The first deposits were added to 75% ethanol to extract Composition II. After filtration, Composition III and Composition IV were acquired by the water extraction-ethanol precipitation method from the second deposits.

The original proportion of each composition was regarded as Level 1 of the orthogonal experiment, no Composition as Level 2, and twice the proportion as Level 3 with the four compositions used as the factors to design the orthogonal compatibility experiment by L_9_(3^4^) in Table 2 and Table 3. Experiments on the nine samples were then performed, followed by anticancer experiments and HPLC analysis.

### 3.3. Anticancer Experiments

#### 3.3.1. Inhibition of Tumor Cells

All nine samples comprising the four compositions (Table 3) were dissolved in 150 mL sterilized distilled water and were gavaged to nine groups of Wistar rats (six in each group) at an equivalent clinical dosage. The dosage of administration was 2 mL/200 g, twice a day, and continuous administration for three days. Three days later, fundus blood from Wistar rats was obtained 2 h after gavage administration to obtain serum in order to investigate the effect of the prescriptions on SKOV3 tumor cell lines [43,44]. After incubation under 5% CO_2_ at 37 °C for 24 h, SKOV3 cells (10^4^ cells per well) were subsequently inoculated onto 96-well plates. After incubation for 12 h, the supernatant was discarded, 100 µL drug serum (10% drug serum + 90% RPMI1640) was added, and 100 µL serum (10% serum + 90% RPMI1640) was added to the control group. After treatment for 24 h, the supernatant was discarded, 100 µL DMSO was added, the 96-well plate was mixed on a microvibrator for an additional 10 min, and the proliferation inhibition rate of SKOV3 tumor cell lines in MTT [3-(4,5-dimethyl-2-thiazolyl)-2,5-diphenyl-2H-tetrazolium bromide] was measured at 490 nm using a microplate reader (THERMO Multiskan MK3 series, Waltham, MA, USA). The experiment was performed in triplicate and an average value was used to determine the final result after the measurement was repeated three times.

The formula used was as follows:

(3) $$W_{n}=1-A_{n}/A_{0}$$

where *W_n_* was the cell proliferation inhibition rate, *A_n_* was the OD value of n-th group, *A*_0_ was the original OD value, and n was one of the nine prescriptions.

#### 3.3.2. Survival Experiment in Tumor-Bearing Nude Mice

SKOV3 cells were suspended in phosphate buffered saline and 1 × 10^70^ cells were inoculated by intraperitoneal (i.p.) injection into 6-week-old female nude mice. After two weeks, the model nude ice were randomly divided into ten groups: model control group and nine prescription treatment groups (six in each group). The model control group of nude mice was administered sterilized distilled water by gavage. All nine prescriptions comprising the four compositions (Table 3) were dissolved in 100 mL sterilized distilled water and were administered by gavage to nine groups of nude mice at an equivalent clinical dosage. The dosage of administration was 0.2 mL/20 g, twice a day, and continuous administration for three weeks. Survival extension was observed, and the survival extension rate was calculated by comparing with the model control group. The prescriptions were administered between meals [45].

The formula used was as follows:

$$S_{n}=\left(D_{0}^{42}-D_{n}^{42}\right)+\left(D_{0}^{48}-D_{n}^{48}\right)+\left(D_{0}^{54}-D_{n}^{54}\right)+\left(D_{0}^{60}-D_{n}^{60}\right)/4$$

where Sn was the survival extension rate. $D_{0}^{42}$, $D_{0}^{48}$, $D_{0}^{54}$, $D_{0}^{60}$ were the death rates on the observation day in the reference group, $D_{0}^{42}$, $D_{0}^{48}$, $D_{0}^{54}$, $D_{0}^{60}$ were the rates in the n-th group and n was one of the nine prescriptions.

### 3.4. Analysis of HPLC Fingerprints

For the analysis of multiple constituents in LCSSY, a Waters 2695 HPLC system was equipped with a Waters 2996 Photodiode Array (PDA) detector (Waters Corporation, Milford, MA, USA) and an Alltech 2000 Evaporative Light-scattering Detector (ELSD) (Alltech Associates, Waukegan, IL, USA), connected to Waters Empower 2.0 Software.

#### 3.4.1. Preparation of Sample Solutions

The samples were accurately weighed and dissolved in ethanol for chromatographic condition I and in methanol for chromatographic condition II, respectively, and then filtered through a 0.22 µm micropore film to yield the sample solution for HPLC to obtain the HPLC fingerprints.

#### 3.4.2. HPLC Conditions

Chromatographic condition I (HPLC-PDA). A Kromasil C18 column (250 mm × 4.6 mm, 5 µm) was used for all analyses and maintained at 30 °C. The mobile phase was a 0.1% formic acid water solution (A) and methanol (B) system. The gradient elution profile was as follows: 0–10 min A:B (50:50, *v*/*v*), 10–25 min A:B (50:50, *v*/*v*) to A:B (35:65, *v*/*v*), 25–30 min A:B (35:65, *v*/*v*), 30–40 min A:B (35:65, *v*/*v*) to A:B (20:80, *v*/*v*), 40–45 min A:B (20:80, *v*/*v*), 45–55 min A:B (20:80, *v*/*v*) to A:B (15:85, *v*/*v*), 55–70 min A:B (15:85, *v*/*v*) to A:B (5:95, *v*/*v*), 70–80 min A:B (5:95, *v*/*v*) to A:B (0:100, *v*/*v*). The flow rate was 1.0 mL/min and the effluent was monitored at 210 nm with the sample injection volume of 10 µL.

Chromatographic condition II (HPLC-PDA-ELSD). A Kromasil C18 column (250 mm × 4.6 mm, 5 µm) was used for all analyses and maintained at 30 °C. The mobile phase was a 0.05% formic acid water solution (A) and methanol solution (B) system. The gradient elution profile was as follows: 0–15 min A:B (95:5, *v*/*v*) to A:B (80:20, *v*/*v*), 15–30 min A:B (80:20, *v*/*v*), 30–55 min A:B (80:20, *v*/*v*) to A:B (55:45, *v*/*v*), 55–60 min A:B (55:45, *v*/*v*) to A:B (20:80, *v*/*v*), 60–70 min A:B (20:80, *v*/*v*) to A:B (5:95, *v*/*v*), 70–80 min A:B (5:95, *v*/*v*) to A:B (0:100, *v*/*v*), 80–85 min A:B (0:100, *v*/*v*). The flow rate was 0.8 mL/min and the effluent was monitored at 270 nm with the sample injection volume of 10 µL. The ELSD was then connected to the PDA and set to a drift tube temperature of 103 °C at gain 4 and the N_2_ flow rate was 2.5 L/min.

#### 3.4.3. Validation of Methodology

According to the established HPLC condition programs, the method precision was assessed by the successive analysis of six injections of sample solutions, which had the best peak shapes, responses, and peak resolution among the samples in preliminary experiments, and repeatability was assessed by the successive analysis of six replicates of the same samples, respectively. In addition, the analysis of different time periods in a day (0, 2, 4, 8, 16, 24 h) was used to evaluate the stability of these samples over 24 h. The relative standard deviation (R.S.D.) of the relative retention time and relative peak area of the characteristic peaks were calculated to evaluate the method, respectively.

#### 3.4.4. Determination of Fingerprints

The fingerprints of the nine samples designed by the L_9_(3^4^) orthogonal test were respectively determined by chromatographic condition I (HPLC-PDA) and chromatographic condition II (HPLC-PDA-ELSD).

### 3.5. Analysis of Spectrum-Effect Relationships

The peak areas in the fingerprints analyzed by chromatographic condition I (HPLC-PDA) and II (HPLC-PDA-ELSD) were respectively marked as X_n_, Y_n_, and Z_n_, which were associated with the pharmacological results of W and S and constituted the data matrix for regression analysis and correlation analysis. Finally, the components with correlated relationships to the pharmacological results were calculated by SPSS statistics software (SPSS for Windows 19.0, SPSS International Business Machines Corp., Armonk, NY, USA) to obtain the union and intersection results in both analyses.

#### 3.5.1. Regression Analysis

Regression is a statistical method used to verify the linearity of the non-linear relationship between one or more independent variables and a dependent variable, and was used to study the spectrum-effect relationships [46,47]. In this study, the ENTER and STEPWISE methods in regression were respectively used to obtain the union results.

#### 3.5.2. Correlation Analysis

Correlation is a statistical method used to study the closeness between variables. The correlation coefficients were calculated by the method of bivariate in correlation [48].

#### 3.5.3. Assignments of the Correlated Peaks

##### Comparison of the Fingerprints with Negative Control Samples and Reference Standard Samples

According to the extraction method of LCSSY mentioned in Section 3.2, eight types of negative control samples were prepared including the formula without *Hirudo*, *Epimedii Folium*, *Cervi Cornu Pantotrichum*, *Fritillariae Thunbergii Bulbus*, *Ginseng Radix et Rhizoma*, *Astragali Radix*, *Sparganii Rhizoma*, and *Curcumae Rhizoma*, respectively.

The mixed standard solution A, containing 45.5 µg/mL germacrone, 20.3 µg/mL furandiene, and 70.9 µg/mL β-elemene was prepared by adding an accurate amount of each standard stock into a volumetric flask and was then dissolved with 10 mL ethanol for chromatographic condition I (HPLC-PDA).

The mixed standard solution B, containing 10.0 µg/mL calycosin-7-glucoside, 7.8 µg/mL ononin, 8.2 µg/mL epimedin B, 10.0 µg/mL icariin, 32.6 µg/mL ginsenoside Rc, 50.0 µg/mL astragaloside, 34.0 µg/mL ginsenoside Rd, and 50.0 µg/mL astragaloside II was prepared by adding an accurate amount of each standard stock into a volumetric flask and was then dissolved with 10 mL methanol for chromatographic condition II (HPLC-PDA-ELSD).

The negative control samples and reference standard samples were prepared and injected into the HPLC system to assign the correlated peaks. The chromatographic conditions used were the same as those described in Section 3.4.2. HPLC analysis.

##### HPLC-MS Analyses

To identify the structure of the correlated peaks, HPLC-MS analyses were performed on a TRAP Mass Spectrometer (LCQ, Finnigan MAT, San Jose, CA, USA) with a Waters 2695 HPLC system (Waters Corporation). The column and elution conditions used were the same as those described in Section 3.4.2. High purity nitrogen (N_2_) was used as the collision gas and nebulizer, respectively. The parameters in negative/positive ion modes were as follows: ion spray voltage, −3.5 kV/3.5 kV; capillary temperature, 350 °C; vaporizer temperature, 35 °C. Spectra were recorded in the range of *m*/*z* 100–1500 for full scan data. The representative sample was prepared and injected into the HPLC-MS system to identify the structure of the correlated peaks.

## 4. Conclusions

Multi-dimensional HPLC fingerprints and pharmacological experiments were conducted to determine the bioactive components related to the anti-ovarian cancer activities of LCSSY. The results suggested that the pharmacodynamic basis of the anti-ovarian cancer activities of LCSSY were X_5_ (unknown component, from *Ginseng Radix et Rhizoma*), X_9_ (germacrone, from *Curcumae Rhizoma*), X_11_ (furandiene, from *Curcumae Rhizoma*), X_12_ (unknown component, from *Curcumae Rhizoma*), X_16_ (β-elemene, from *Curcumae Rhizoma*), X_18_ (unknown component, from *Sparganii Rhizoma*), Y_5_ (calycosin-7-glucoside, from *Astragali Radix*), Y_8_ (ononin, from *Astragali Radix*), Y_12_ (epimedin B, from *Epimedii Folium*), Y_14_ (icariin, from *Epimedii Folium*), Y_20_ (unknown component, from *Fritillariae Thunbergii Bulbus*), Z_4_ (ginsenoside Rc, from *Ginseng Radix et Rhizoma*), Z_5_ (astragaloside, from *Astragali Radix*), Z_6_ (ginsenoside Rd, from *Ginseng Radix et Rhizoma*), and Z_8_ (astragaloside II, from *Astragali Radix*), in which, X_11_ (furandiene, from *Curcumae Rhizoma*), X_12_ (unknown component, from *Curcumae Rhizoma*), X_16_ (β-elemene, from *Curcumae Rhizoma*), X_18_ (unknown component, from *Sparganii Rhizoma*), Y_5_ (calycosin-7-glucoside, from *Astragali Radix*), Y_12_ (epimedin B, from *Epimedii Folium*), and Z_5_ (astragaloside, from *Astragali Radix*) were the most closely correlated peaks.

## Figures and Tables

**Table 1:** Assignments of the correlated peaks.

No.	Mass Data		Compound	Formula	Mol. Wt.	Assignment
X_5_		268	Unknown	-	*-*	*Ginseng Radix et Rhizoma*
X_9_	[M + H]^+^	219	germacrone	C_15_H_22_O	218	*Curcumae Rhizome*
X_11_	[M + H]^+^	217	furanodiene	C_15_H_20_O	216	*Curcumae Rhizome*
X_1__2_		285	Unknown	-	*-*	*Curcumae Rhizome*
X_1__6_	[M + H]^+^	205	β-elemene	C_15_H_24_	204	*Curcumae Rhizome*
X_1__8_		180	Unknown	-	*-*	*Sparganii Rhizoma*
Y_5_	[M + H]^+^	447	calycosin-7-glucoside	C_22_H_22_O_10_	446	*Astragali Radix*
	[M + H − glc]^+^	285				
Y_8_	[M + H]^+^	431	ononin	C_16_H_13_O_4_	430	*Astragali Radix*
	[M + H − glc]^+^	269				
Y_12_	[M + H]^+^	809	epimedin B	C_38_H_48_O_19_	808	*Epimedii Folium*
	[M + H − xyl]^+^	677				
	[M + H – xyl − rha]^+^	531				
Y_14_	[M + H]^+^	677	icariin	C_33_H_40_O_15_	676	*Epimedii Folium*
	[M + H − rha]^+^	531				
Y_20_		442	Unknown	-	-	*Fritillariae Thunbergii Bulbus*
Z_4_	[M − H]^−^	1077	Ginsenoside Rc	C_53_H_90_O_22_	1078	*Ginseng Radix et Rhizoma*
Z_5_	[M – H + HCOOH]^−^	829	Astragaloside	C_41_H_68_O_14_	784	*Astragali Radix*
Z_6_	[M – H + HCOOH]^−^	991	Ginsenoside Rd	C_48_H_82_O_18_	946	*Ginseng Radix et Rhizoma*
Z_8_	[M – H + HCOOH]^−^	871	Astragaloside II	C_43_H_70_O_15_	826	*Astragali Radix*

**Table 2:** Factors and levels selected.

Factor Level	Composition I(g) A	Composition II(g) B	Composition III(g) C	Composition IV(g) D
1	1(6.60)	1(22.46)	1(2.70)	1(8.33)
2	0	0	0	0
3	2(13.20)	2(44.92)	2(5.40)	2(16.66)

**Table 3:** Array of orthogonal compatibility experiments.

Test	A(g)	B(g)	C(g)	D(g)
1	1(6.60)	1(22.46)	1(2.70)	1(8.33)
2	1(6.60)	0	0	0
3	1(6.60)	2(44.92)	2(5.40)	2(16.66)
4	0	1(22.46)	0	2(16.66)
5	0	0	2(5.40)	1(8.33)
6	0	2(44.92)	1(2.70)	0
7	2(13.20)	1(22.46)	2(5.40)	0
8	2(13.20)	0	1(2.70)	2(16.66)
9	2(13.20)	2(44.92)	0	1(8.33)

## Abbreviations

The following abbreviations are used in this manuscript.

LCSSY	Lichong Shengsui Yin
TCMs	Traditional Chinese medicines
HPLC-PDA	High-performance liquid chromatography-photodiode array detector
HPLC-PDA-ELSD	High-performance liquid chromatography-photodiode array detector -Evaporative Light-scattering Detector
HPLC-MS	High-performance liquid chromatography-mass spectrometry detector
HPTLC	High performance thin layer chromatography
GC-MS	Gas chromatography-mass spectrometry
IR	Infrared spectroscopy
HPCE	High performance capillary electrophoresis
R.S.D.	Relative standard deviation
MTT	3-(4,5-dimethyl-2-thiazolyl)-2,5-diphenyl-2H-tetrazolium bromide
i.p.	Intraperitoneal injection
